# Association Proceedings

**Published:** 1902-10

**Authors:** 


### ASSOCIATION PROCEEDINGS.

### INTERSTATE ASSOCIATION OF SANITARY BOARDS.

### *Report of Committee on Line and Open Season.*

WICHITA, KANSAS, September 23, 1902.

TO HONORABLE W. H. DUNN, PRESIDENT:

We, your Committee on Line and Open Season, beg leave to report as follows:

1. We recommend to the Department of Agriculture that the national quarantine line remain the same as last year, with the exception that the counties of Moore and Bledsoe, in the State of Tennessee, be placed above said line after being examined and recommended by an agent of the Bureau of Animal Industry.

2. We recommend that cattle from below the Federal quarantine line be allowed to be moved to points above said line within the States of Texas and Kansas and the Territory of Oklahoma between November 1 and December 31, 1902, and to points within the States of Virginia, North Carolina, Tennessee, and Missouri, and the Territories of Arizona and New Mexico between November 1, 1902, and January 31, 1903, under the sanitary regulations provided by these States and Territories and permitted by the local authorities in charge, provided that no such movement of Southern cattle shall be allowed into any of these States or Territories where proper local regulations are not enforced.

3. All cattle from the quarantine district destined to points outside of the States and Territories above named may be shipped without inspection between November 1, 1902, and January 31, 1903, inclusive, and without restrictions other than may be enforced by local regulations at point of destination.

4. We recommend that cattle from the two northern tiers of counties in Arkansas be admitted into the State of Missouri by inspection from February 1 to March 31, 1903.

Respectfully submitted,

DR. J. C. NORTON,
Chairman.

M. M. HAWKINS,
Secretary.

### AMERICAN VETERINARY MEDICAL ASSOCIATION.

*Committees,* 1902 *and* 1903.

*Executive.*—Tait Butler, J. F. Winchester, H. L. Ramacciotti, F. Torrance, S. Brenton, W. Horace Hoskins, A. H. Baker.

*Finance.*—J. E. Ryder, B. McInnes, F. Abele.

*Publication.*—M. H. Reynolds, R. R. Bell, R. P. Lyman, C. J. Marshall, C. W. Gay.

*Intelligence and Education.*—E. B. Ackerman, A. T. Peters, W. J. Hinman, Paul Fischer, E. M. Ranck.

*Diseases.*—L. Pearson, V. A. Moore, S. D. Brimhall, L. Frothingham, R. R. Dinwiddie.

*Army Legislation.*—W. H. Lowe, Wm. Dougherty, Austin Peters, M. E. Knowles, Wm. H. Kelly.

*Resolutions.*—D. E. Salmon, G. A. Johnson, N. S. Mayo, G. R. White, Alexander Burr.

*Pharmacopœia.*—L. A. Merillat, R. R. Bell, D. King Smith, E. L. Quitman, J. J. Repp, E. M. Ranck, H. D. Hanson.

---

### P. S. V. M. A. DOINGS.

President Rhoads suggested many salient thoughts in his semi-annual address, and offered some field of work for every member of the Association.

The meeting was not as strong in papers offered as usual, but was rich in valuable talks and discussions of live subjects by those who were present.

President Sallade, of the State Board of Veterinary Medical Examiners, was there with his arm in a sling from a broken clavicle eager to discuss a new registration law of veterinarians in the State.

Everybody was pleased to find Dr. Noack, president of the Schuylkill Valley Veterinary Medical Association, in improved health and so active in the pleasant entertainment afforded the visiting veterinarians.

The veterinary poet of Allentown was much in evidence at the semi-annual meeting of the Association.

Everybody was pleased to see our aggressive sister association of New Jersey represented by one of its faithful members, Dr. L. P. Hurley, of Hopewell.

The "Good Roads" meeting inaugurated by President Rhoads proved a very interesting and instructive gathering. The lessons taught through stereopticon screen presentations of this live subject of the day were very impressive indeed, and awakened a deeper interest in the subject in all those who were privileged to attend.

